# Understanding Older Adult's Technology Adoption and Withdrawal for Elderly Care and Education: Mixed Method Analysis from National Survey

**DOI:** 10.2196/jmir.7401

**Published:** 2017-11-03

**Authors:** Ching-Ju Chiu, Chia-Wen Liu

**Affiliations:** ^1^ Institute of Gerontology, College of Medicine, National Cheng Kung University Tainan Taiwan; ^2^ Institute of Physical Therapy, College of Medicine, National Cheng Kung University Tainan Taiwan

**Keywords:** Internet adoption, Internet withdrawal, digital opportunity, information literacy, middle-aged and older adults, Taiwan

## Abstract

**Background:**

Elderly adults have comprised the fastest growing population adopting the Internet and computer technology over the past decade. However, how their experiences can shed light on elderly learning theory has not been examined much in the literature.

**Objective:**

This study investigated the factors and reasons associated with Internet adoption and withdrawal among older adults in Taiwan, and if any gender differences exist in this context.

**Methods:**

Data on participants aged 50 years and older from the nationally representative “Digital Opportunity Survey on Individuals and Households in Taiwan,” who did not use the Internet in 2005 but adopted it in 2007 (n=1548), and those who reported using Internet in 2011 but then withdrew (n=1575), were analyzed. Factors and reasons associated with Internet adoption and withdrawal were examined using both quantitative and qualitative data.

**Results:**

Education level independently predicted Internet adoption behavior. With regard to the reasons for adoption, 66% (62/94) of participants indicated they started using the Internet to meet certain “needs”; for example, “keeping up with the world” (40.4%, 38/94) was listed as the most critical reason, followed by “job needs” (25.5%, 24/94). Older adults with a positive attitude toward the Internet with regard to increasing employment opportunities (OR 2.0, 95% CI 1.0-3.9, *P*=.04) and the amount of information obtained (OR 0.5, 95% CI 0.3-0.9, *P*=.01), as well as enriching recreation and entertainment (OR 0.6, 95% CI 0.4-0.9, *P*=.02), were less likely to withdraw from the Internet. The most common reason for Internet withdrawal was “psychological barriers” (eg, no available time, no meaningful use, or nothing worth reading/watching; 66.3%, 193/291), followed by “health barriers” (eg, eyes or body deteriorate with Internet use; 21.0%, 61/291). Although psychological barriers were the most important factor for Internet withdrawal for both men (72.5%, 100/138) and women (62%, 93/150), women were more likely than men to be affected by health barriers (26.0%, 39/150 vs 15.9%, 22/138; *P*=.004) and anthropic factors or accidental barriers (7.3%, 11/150 vs 2.9%, 4/138; *P*=.02).

**Conclusions:**

Our findings that the need to keep up with the world associated with Internet adoption, and gender differences in reasons behind Internet withdrawal, such that women reported more health and anthropic factors or accidental barriers than man, may provide a new perspective that help health educators understand strategies that encourage older adults to keep learning, an important component of active aging.

## Introduction

Demographic statistics indicate that 8.1% of the global population were older adults in 1960, and this number grew to 10% in 2000, with estimates suggesting that 21.4% of the population will be senior citizens in 2050 [1]. A demographic survey in the United States indicated that the elderly population will increase from 13% in 2010 to 19% in 2030 [2]. Internet use is increasingly widespread, although the number of people who do not use the Internet frequently, such as the elderly, is in fact increasing [3]. Despite this, the first nationally representative study in the United States showed that the proportion of seniors (those aged 65 years and older; mean 75, SD 7.4 years) who use any digital health technologies significantly increased from 21% in 2011 to 25% in 2014 [4].

Internet use can bring many benefits, such as increasing the happiness and decreasing the loneliness of older adults in a retirement community [5], encouraging social connections, and accelerating information exchanges among adults older than 50 years in the United States [6]. Studies have shown that the social functions that come from Internet use contribute to the maintenance of relationships for older adults aged 57 to 87 years [7], particularly for older people with limited mobility, because it is a good way for them to come out of solitude and reconnect to the rest of the world [6]. One of the significant aims of active aging is to keep learning in order to adapt to the changes that occur in later life, gain capabilities similar to those of younger people, and remain productive [8], and it is believed that older adults’ involvement in the Internet may achieve this [9-13]. But the Internet is associated with security concerns and learning anxiety (eg, how to handle the operating systems, how to resolve any problems encountered, and the fear that one might break an expensive device) [14,15]. Not every population group benefits from Internet use, and there can even be certain negative impacts. However, many studies in the United States indicate that there are more positive than negative impacts arising from Internet use [16-18].

Many studies have been conducted on why older people may choose to avoid the Internet, such as the webpage design not being suitable for older people and the perception of not being able to learn new things because of insufficient cognitive capability, vision, or motor function [19-27]. The diverse needs of older users should be considered in the design and development of such technology [13]. However, there are still only a handful of studies that aim to understand Internet withdrawal among middle-aged and older adults. A previous study showed that the objective of e-learning was not only to describe something, but also to demonstrate how to do it [28]. Knowles’ adult education theory [29] highlights the importance of self-direction, a reservoir of experience, social roles, and problem-centered orientation in affecting adult learning, and Internet adoption and withdrawal may be affected by these factors. For example, the Internet creates an independent learning opportunity for the self-directed learning of adults, while family or friends can serve as a facilitator. Older learners have a large body of experience to serve as a background for new learning, and they are strongly motivated to learn information that has immediate application [30]. In contrast, if adults feel they are unable to learn, they are more likely to withdraw from Internet use.

Gender differences may also exist in Internet adoption and withdrawal among middle-aged and older adults. Previous studies indicated that website attributes [31], Internet usage patterns [32], and habits [33] are different across genders. More males use the Internet than females [32], and the differences in their visual cues have a great impact on their online choices [34]. Women have also expressed greater levels of anxiety toward computers [35], less self-perceived competence, and lower perceived ease of use with respect to the Internet [36] than men. Moreover, older males seem to perceive the Internet as more useful due to their perceived higher levels of ease of use than females [37]. However, some studies found no differences across genders in these respects [38,39]. It is thus an interesting issue whether there are any gender differences in Internet adoption and withdrawal. If we could underline different characteristics associated with gender, it would encourage the development of genuinely usable information and communications technology products, training, and support approaches, and it could be used as a basis for the design of continuing education materials or for market segmentation of men and women.

In Taiwan, there is little data on older adults’ Internet use, with no nationwide surveys. As a result, there is no solid reference material for Internet adoption and withdrawal among middle-aged and older adults in Taiwan. In addition, because older adults are the fastest growing population in adopting the Internet and computer technology during the past decade [40], depicting factors and reasons associated with Internet adoption and withdrawal may shed light on some aspects of elderly learning theory. Thus, the purposes of this study were (1) to understand the prevalence of Internet adoption and withdrawal among middle-aged and older adults in Taiwan, (2) to examine the factors associated with Internet adoption and withdrawal among middle-aged and older adults in Taiwan, (3) to identify the reasons behind those who had Internet adoption and withdrawal, and (4) investigate if the aforementioned patterns differed by gender.

## Methods

### Study Participants and Data Sources

Data were derived from an on-going survey, the Individual & Household Digital Opportunity Survey, approved by the National Development Council in its Research on Constructing the Index System of Digital Opportunity Development in Taiwan. It covers home phone users and interviews native Taiwan citizens aged 12 years or older residing in ordinary households, and it has been carried out every year since 2002. Computer-assisted telephone interviews were adopted in this survey using a random stratified sampling procedure.

Each year’s survey mainly covers information on Internet access, information literacy, information application, digital opportunity, and digital exclusion. Each year’s survey is designed as a cross-sectional study of that year. However, only one follow-up survey was conducted, with the first and second surveys taking place in 2005 and 2007. Those participants who did not use the Internet in 2005 were asked again in 2007 whether they had done so. As a result, we used the data in 2007 for the issue of Internet adoption. In addition, only the 2011 survey asked a question about Internet withdrawal; therefore, the 2011 survey was selected to examine the issue of Internet withdrawal. A total of 1548 valid participants aged 50 years and older in 2007 (success rate: 72.4%) and 1575 adults in 2011 (success rate: 69.4%) were analyzed in this study.

### Measures

#### Sociodemographic Variables

Age (range 51-94 years), gender, living area (northern, central, southern, and eastern Taiwan and outer islands), economic status (with an income of Taiwanese new dollar [NT$] NT$30,000 or less, between NT$30,000 and NT$90,000, and NT$90,000 or more per month), and educational background (elementary school or lower, high school, college or higher) were recorded.

#### Internet Adoption/Withdrawal

In this study, those who did not use the Internet in 2005 were asked in 2007 whether they now did. Those who answered “yes” were defined as Internet adopters, whereas those who answered “no” were defined as Internet nonadopters. For Internet withdrawal, the definition was based on the Internet use experience in 2011 and whether the person had used the Internet in the last month. Those who had previous experience of Internet use and had used it in the last month were defined as Internet nonwithdrawers, whereas those who had previous experience of Internet use but had not used it for a month were defined as Internet withdrawers. The reasons for Internet adoption and withdrawal were obtained from an open-ended question. Participants were asked: “Why did you start to use the Internet?” in 2007 and “Why don’t you use the Internet anymore?” in 2011.

#### Opinions on Internet Use

Opinions on Internet use were divided into three dimensions of “perceived helpfulness,” “perceived fun,” and “perceived interpersonal interaction.” The question for perceived helpfulness was “Is Internet use in your opinion helpful to your life?” and the choices were 1=very helpful, 2=somewhat helpful, and 3=not helpful. The question for perceived fun was “Do you feel that Internet use gives you more fun, less fun, or no effect at all?” and the choices were 1=less fun, 2=no effect, and 3=more fun. The question for perceived interpersonal interaction was “Does the Internet allow you to interact with your friends and relatives more frequently, less frequently, or have no effect at all?” and the choices were 1=more frequently, 2=less frequently, and 3=no effect.

#### Digital Opportunity

“Digital opportunity” referred to whether the respondents felt any change or opportunities created from starting to use the Internet in their daily lives (eg, opportunities for employment and learning, or changes in their circle of friends or income). The study participants were asked if they had noticed any of the following changes: “Has your circle of friends / employment opportunity / learning opportunity / income / access to useful information / recreation and entertainment / government information increased because of your Internet use?” and “Is it possible for you to connect with others who share the same views in politics or policies?” (eight questions in total). The choices were 1=yes and 2=no.

#### Information Literacy

“Information literacy” referred to the basic capabilities that are required for Internet use (eg, becoming a member of specific website; downloading and uploading files, video clips, or photos). The study participants were asked, “Have you applied for user accounts and passwords and become a member of specific website?” and “Do you know how to download and upload files, video clips, or photos?” The choices were 1=yes and 2=no. They were also asked, “Are you familiar with any kind of word processing program (eg, Word, Notepad, or Writer) for document editing?” and the choices were 1=very familiar, 2=somewhat familiar, 3=not very familiar, and 4=no idea whatsoever. The distribution of information literacy was determined based on these categories.

### Statistical Analysis

To examine the sociodemographic characteristics, opinions for Internet use, digital opportunity, and information literacy between participants with and without Internet adoption or withdrawal, *t* tests and chi-square tests were used for the ordinal and nominal variables, respectively. Second, logistic regression analysis was employed to examine independent factors predicting Internet adoption and withdrawal with comparison to those individuals without adoption or withdrawal. Third, open data coding was carried out for the reasons for Internet adoption and withdrawal, with the results described using the frequency and percentage. We finally examined gender differences with regard to the aforementioned patterns, stratifying the analysis by gender.

## Results

### Sociodemographic Characteristics and Opinions on Internet Use, Digital Opportunity, and Information Literacy

The distribution of demographic characteristics and opinions about Internet use, digital opportunity, and information literacy of the participants with and without Internet adoption or withdrawal is presented in Table 1. Among the 1548 participants in 2007, only 6.65% (n=103) adults belonged to the Internet adoption group (nonadopters: 1445/1548, 93.35%). Among the 1575 adults in 2011, only 18.60% (n=293) belonged to the Internet withdrawal group (nonwithdrawers: 1282/1575, 81.40%). When it came to adopting the Internet, the factors of age, educational background, family income per month, and influence on life were significantly different between the adopters and nonadopters, whereas gender was not. Most of the Internet adopters had a high school or above diploma (89/103, 86.4%); nearly 60% (60/103) of the adopters had a family income of more than NT$30,000 per month. Most of the adopters believed the Internet helped with their lives (85/103, 85.9%), and 60.2% (62/103) felt that it introduced more fun in their lives.

**Table 1:** Sociodemographic characteristics, opinions on Internet use, digital opportunity, and information literacy between participants who did and did not adopt the Internet or withdraw from the Internet.

Item by Internet adoption or withdrawal			Yes (adopted/withdrew)	No (did not adopt/withdraw)	*P*
**Internet adoption**			**N=103**	**N=1445**	
	Age (years), mean (SD)		58.4 (6.0)	65.2 (9.6)	<.001
	Gender (male), n (%)		54 (52.4)	681 (47.1)	.30
	**Education, n (%)**				<.001
		Elementary school or below	14 (13.6)	1043 (72.2)	
		High school	66 (64.1)	333 (23.1)	
		College or above	23 (22.3)	69 (4.8)	
	**Family income/month (NT$), n (%)**				<.001
		<30,000	43 (41.3)	986 (68.2)	
		30,000~90,000	45 (44.0)	384 (26.6)	
		>90,000	15 (14.7)	75 (5.2)	
	**Opinions on Internet use, n (%)**				
		Perceived helpfulness	85 (85.9)	—	—
		Perceived fun	62 (60.2)	—	—
		Perceived more interpersonal interaction	21 (20.4)	—	—
**Internet withdrawal**			**N=293**	**N=1282**	
	Age (years), mean (SD)		59.6 (7.1)	57.6 (5.6)	<.001
	Gender (male), n (%)		139 (47.4)	705 (55.0)	.02
	**Education, n (%)**				<.001
		Elementary school or below	40 (13.7)	58 (4.5)	
		High school	173 (58.9)	524 (40.9)	
		College or above	80 (27.4)	700 (54.6)	
	**Family income/month (NT$), n (%)**				<.001
		<30,000	82 (27.9)	146 (11.4)	
		30,000~90,000	152 (52.1)	563 (43.9)	
		>90,000	59 (20.0)	573 (44.7)	
	**Digital opportunity, n (%)**				
		Larger circle of friends	72 (24.6)	459 (35.8)	.001
		More job opportunities	35 (12.0)	237 (18.5)	.009
		More learning opportunities	123 (41.8)	948 (73.9)	<.001
		Increased income	16 (5.5)	142 (11.1)	.004
		More information of life	120 (41.0)	997 (77.8)	<.001
		Enriched recreation and entertainment	114 (39.0)	914 (71.3)	<.001
		More government information	90 (30.7)	761 (59.4)	<.001
		Ability to connect with others with the same political/policy views	14 (4.6)	213 (16.6)	<.001
	**Information literacy, n (%)**				
		Know how to apply for a user account and password	52 (17.8)	705 (55.0)	<.001
		Know how to use a word processing program	135 (46.1)	885 (69.0)	<.001
		Know how to upload and download files	99 (33.8)	883 (68.9)	<.001

The analysis of Internet withdrawal shows that the factors of age, gender, educational background, family income per month, digital opportunity, and information literacy were significantly different between those who withdrew or did not, whereas where they lived did not. For the withdrawers, most were female (154/293, 52.6%) and 72.6% of them (213/293) had a high school or lower diploma. Approximately 80% of the withdrawers (234/293) had a family income per month of NT$ 90,000 or less. The top three digital opportunities that the nonwithdrawers had were Internet use helps with the “improved learning opportunity” (123/293, 41.8%), followed by “more access to useful information” (120/293, 41.0%), then “enriched recreation and entertainment” (114/293, 39.0%). In addition, less than 50% of the withdrawers (135/293, 46.1%) selected “know how to use a word processing program (eg, Word or Notepad) for document editing” for their information literacy, whereas the percentage was greater than 50% for nonwithdrawers in all three categories of information literacy.

### Factors Predicting Internet Adoption and Withdrawal

The results of the logistic regression analysis evaluating the independent effects of the demographic characteristics, opinions on Internet use, digital opportunity, and information literacy of the study participants on Internet adoption and withdrawal are presented in Table 2. For Internet adoption, the older the participants were, the less likely they were to experience adoption (OR 0.9, 95% CI 0.92-0.9), and similar results for educational background, as those with less education had less adoption. For withdrawal, the Internet withdrawal risk was significantly lower for middle-aged and older adults who had a family income per month of NT$90,000 or more (as opposed to those with NT$30,000 or less; OR 0.4, 95% CI 0.2-0.8), who felt enriched recreation and entertainment (OR 0.6, 95% CI 0.4-0.9), access to more useful information (OR 0.5, 95% CI 0.3-0.9), knew how to apply for a user account and password (OR 0.4, 95% CI 0.2-0.6), and how use a word processor (OR 0.6, 95% CI 0.4-0.9). On the other hand, the risk of Internet withdrawal was significantly higher for those participants who were older (OR 1.1, 95% CI 1.0-1.1).

**Table 2:** Factors predicting Internet adoption and withdrawal among middle-aged and older adults by logistic regression.

Factor		Adoption (yes/no)		Withdrawal (yes/no)	
		OR (95% CI)	*P*	OR (95% CI)	*P*
Age		0.9 (0.9-0.9)	.001	1.1 (1.0-1.1)	.003
Gender (male/female)		0.8 (0.4-1.3)	.28	0.7 (0.4-1.0)	.07
**Educational background**					
	High/elementary school or below	10.0 (4.9-20.4)	<.001	0.9 (0.4-2.0)	.89
	College or above/elementary school or below	19.4 (7.9-47.1)	<.001	0.5 (0.2-1.1)	.10
**Family income/month (NT$)**					
	30,000-90,000/<30,000	0.9 (0.5-1.7)	.85	0.8 (0.5-1.4)	.39
	>90,000/<30,000	1.0 (0.4-2.4)	.10	0.4 (0.2-0.8)	.008
**Digital opportunity (yes/no)**					
	larger circle of friends	—	—	1.4 (0.9-2.3)	.19
	More job opportunities	—	—	2.0 (1.0-3.9)	.04
	More learning opportunities	—	—	0.8 (0.5-1.3)	.31
	Increased income	—	—	0.5 (0.2-1.4)	.17
	More life information	—	—	0.5 (0.3-0.9)	.01
	Enriched recreation and entertainment	—	—	0.6 (0.4-0.9)	.02
	More government information	—	—	0.8 (0.5-1.2)	.28
	Ability to connect with others who have the same political/policy views	—	—	0.6 (0.3-1.4)	.24
**Information literacy (yes/no)**					
	Know how to apply for a user account and password	—	—	0.4 (0.2-0.6)	<.001
	Know how to use a word processing program	—	—	1.2 (0.8-1.9)	.43
	Know how to upload and download files	—	—	0.6 (0.4-0.9)	.03

**Table 3:** Reasons for Internet adoption and withdrawal for middle-aged and older adults.

Type and item			n (%)	
**Internet adoption**			**N=94**
	**Needs (66.0%)**	
		Job needs	24 (25.5)
		Keep up with the world	38 (40.4)		
	**Expansion of life (24.4%)**	
		Look for data and information	9 (9.6)
		Stock market	6 (6.4)
		Interested in learning	4 (4.3)
		Online ticket purchase	2 (2.1)
		Read news	2 (2.1)
	**Recreation and entertainment (9.6%)**	
		Play games and have fun	4 (4.3)
		Kill some time	4 (4.3)
		Watch TV online	1 (1.1)
**Internet withdrawal**^a^			**N=291**
	**Health barriers (21.0%)**	
		I am old and my eyes or body has deteriorated	48 (16.5)
		I forgot how it works; it does not work well for me	13 (4.5)
	**Psychological barriers (66.3%)**	
		I do not have the time	128 (44.0)
		It has no use for me	53 (18.2)
		Nothing worth watching/reading	12 (4.1)
	**Equipment or environmental barriers (7.2%)**	
		The computer is out of order	10 (3.4)
		I do not have a computer or Internet access at home	8 (2.8)
		Internet access is too expensive	1 (0.3)
		Internet connection is poor	1 (0.3)
		Stop for home renovation	1 (0.3)
	**Anthropic factor or accidental barriers (5.5%)**	
		Others need the computer at home	8 (2.8)
		Travel to a foreign country or stay somewhere else for a period of time	3 (1.0)
		Family accident	2 (0.7)
		Internet access is canceled so that kids will not use it too much	2 (0.7)
		Retirement	1 (0.3)

^a^The sample size of Internet withdrawers was 293 adults and there were two with missing data; therefore, the final sample size of Internet withdrawers was 291 older adults.

### Reasons for Internet Adoption and Withdrawal

Table 3 shows the results of the causes for middle-aged and older Internet adopters and withdrawers. For the reason of adoption, 66% (62/94) of participants indicated they started using Internet “out of need,” followed by “expansion of life” (23/94, 24.4%), and then “recreation and entertainment” (9/94, 9.6%). Among “needs,” “keep up with the world” (38/94, 40.4%) was listed as the most critical reason, followed by “job needs” (24/94, 25.5%). For the reason of withdrawal, the highest percentage went to “psychological barriers,” such as having no time available, no meaningful use, or nothing worth reading/watching (193/291, 66.3%); followed by “health barriers” such as eyes or body deteriorating or do not work well enough to use the Internet (61/291, 21.0%), “equipment barriers” such as having no computer or Internet access at home (21/291, 7.2%), and “anthropic factor or accidental barriers” such as having no computer to use because others need it at home, or travel to a foreign country for a period of time (16/291, 5.5%).

### Gender Differences

The results of the analysis for the reasons for Internet withdrawal among the middle-aged and older adults by gender are shown in Table 4. For the details of withdrawal reason, a large proportion of Internet withdrawals could be attributed to having no time available (males: 67/138, 44.7%; females: 61/150, 44.2%). However, for women the second most significant reason for withdrawal was “I am old and my eyes or body has deteriorated” (32/150, 21.3%) and the third was “it has no use for me” (21/150, 14.0%). For men, the second most significant reason for withdrawal was “it has no use for me” (32/138, 23.2%) and the third was “I am old and my eyes or body has deteriorated” (16/138, 11.6%). For the category of withdrawal reason, the motivation for withdrawal was significantly more often due to anthropic factor or accidental barriers (11/150, 7.3% vs 4/138, 2.9%, *P*=.02) or health barriers (39/150, 26.0% vs 22/138, 16.0%, *P*=.004) for women than for men. Moreover, despite the failure to reach statistical significance, men had more psychological and equipment-related barriers than women. Nevertheless, it was found that the psychological barriers were the most important factor influencing Internet withdrawal for both males and females. A total of 72.5% (100/138) males and 62.0% (93/150) females believed that their Internet withdrawal behaviors were associated with psychological factors such as having no time available, no meaningful use, or nothing worth watching/reading.

**Table 4:** Reasons for Internet withdrawal among middle-aged and older men (n=138) and women (n=150).

Reason for Internet withdrawal			Men, n (%)		Women, n (%)		*P*	
**Reasons (by “details”)**								
	**Health barriers**							
		I am old and my eyes or body has deteriorated		16 (11.6)		32 (21.3)		.003
		I forgot how it works; it does not work well for me		6 (4.4)		7 (4.7)		.52
	**Psychological barriers**							
		I do not have the time		61 (44.2)		67 (44.7)		.11
		It has no use for me		32 (23.2)		21 (14.0)		.44
		Nothing worth watching/reading		7 (5.1)		5 (3.3)		.82
	**Equipment or environmental barriers**							
		The computer is out of order		6 (4.4)		4 (2.7)		.75
		I do not have a computer or Internet access at home		5 (3.6)		3 (2.0)		.67
		Internet access is too expensive		1 (0.7)		0 (0.0)		.37
		Internet connection is poor		0 (0.0)		1 (0.7)		.27
		Stop for home renovation		1 (0.7)		0 (0.0)		.37
	**Anthropic factor or accidental barriers**							
		Others need the computer at home		1 (0.7)		7 (4.7)		.02
		Travel to a foreign country or stay at somewhere else for a period of time		0 (0.0)		3 (2.0)		.06
		Family accident		2 (1.5)		0 (0.0)		.20
**Reason (by “category”)**								
	Health barriers		22 (16.0)		39 (26.0)		.004	
	Psychological barriers		100 (72.5)		93 (62.0)		.41	
	Equipment or environmental barriers		12 (8.7)		7 (4.7)		.53	
	Anthropic factor or accidental barriers		4 (2.9)		11 (7.3)		.02	

## Discussion

This study is the first to use nationally representative data for older adults in Taiwan to examine the issues of Internet adoption and withdrawal. The results showed that both the respondents who adopted the Internet and with greater probability of continued use had the characteristics of higher proportion in men, younger and with higher education. In addition, the most important reasons for Internet adoption were associated with “needs,” especially keeping up with the world and job needs. Furthermore, although psychological barriers were the most important factor of Internet withdrawal for both men and women, women were more likely to be affected by health and anthropic factors or accidental barriers in this regard.

A number of factors associated with older adults’ technology adoption have been documented in the literature [41,42], although the reasons behind these factors have rarely been examined. This study found that the main reasons why older adults may choose to use the Internet are associated with needs, especially with regard to keeping up with the world and job-related needs. In addition, if older adults think that using the Internet can increase their employment opportunities (OR 2.0, 95% CI 1.0-3.9, *P*=.04), access to useful information (OR 0.5, 95% CI 0.3-0.9, *P*=.01), and recreation and entertainment, then they are less likely to withdraw from using it. These findings echo previous research indicating that older adults who do not use new technology or learn new things may simply not see the need for much of what is being offered [43]. Education or training should be provided to help older adults understand the underlying structures and benefits of new learning opportunities.

Many of the existing studies focused on how deteriorating health conditions can hinder older adults’ use of technology or learning efforts. Recent international studies also indicated that people tend to withdraw from the Internet due to health-related factors (eg, age-related changes in visual acuity, color perception and susceptibility to glare, and hearing problems) and some psychological barriers (eg, computer anxiety, online problems, and privacy issues) [15,16,44-46]. This study used a nationally representative sample and the results echo those of other recent works, showing that psychological barriers play the most important role in Internet withdrawal among middle-aged and older adults in Taiwan, followed by health factors. Moreover, it was surprising to find that most of the middle-aged and older adults in this study’s data felt that “no available time” was the main reason that kept them from not using Internet. This suggests that elderly education should not only focus on health conditions because psychological factors may be critical to Internet usage and learning. For example, the selective optimization with compensation model of successful aging [47] may be useful in helping older adults to cope with the barriers they face when adopting new learning. Compensation reminds older adults to consider the reality of a person’s capacities and the health barriers they face, selection refers to the ways older adults use to overcome such barriers, whereas optimization highlights the resources older adults have for achieving their goals.

Gender differences in Internet use have been documented in a number of studies, although in this study using Taiwanese data, men and women were not different in Internet adoption. However, they were different in Internet withdrawal, with men less likely to withdraw from Internet use than women were. In addition, for both men and women, the most common response for the reason for Internet withdrawal was for “psychological barriers” (eg, no time available, no meaningful use, or nothing worth reading/watching) followed by “health barriers” (eg, eyes or body deteriorating with Internet use). Although “psychological barriers” were the most important factor for Internet withdrawal among both men and women, women were more likely than men were to be affected by health and anthropic factors or accidental barriers. We suggest that in addition to focusing on health barriers, such as relearning to overcome cognitive declines, it is also important to work to reduce anthropic factors or accidental barriers, possibly by providing more resources, to encourage women to use new technology or take more learning opportunities.

This study has some limitations. First, this work was based on a cross-sectional survey and it may not be possible to draw any conclusions on the causal relationship between Internet user types and social engagement. Second, due to our data structure we only investigated Internet adoption and withdrawal behavior during 2007 and 2011. Given the rapidly changing nature of the Internet and technology adoption by older adults, we acknowledge that the dynamics of adoption and withdrawal may change over time. However, although the next generation of older adults will have extensive Internet experience, different problems and solutions may arise with regard to adoption and withdrawal behaviors due to the nature of the human learning process. Moreover, the rapid pace of technological change means that “future older generations” are likely to confront an array of technologies they little understand and generally find inaccessible. According to Hanson [48], understanding the general technology-related skills of older users, identifying the strategies successfully used by this population, and finding designs that are optimized for older adults’ abilities (eg, life experiences and knowledge) are the most promising directions for research into technology or computer use by older adults. We believe that factors and reasons associated with Internet adoption and withdrawal found in this study can help to inform education or care for the elderly, a subpopulation that is not in the mainstream or main working force of a nation, and who have special needs associated with their physical and psychological degeneration. Third, due to the limitations of secondary data, this study only examined basic sociodemographic correlates. Various factors that may be related to Internet adoption or withdrawal, such as having a disability or living alone, should be examined in future research. Fourth, the definition of Internet withdrawal remains unclear in the literature. In this study, we defined it as if those participants with Internet experience had not used the Internet during the previous month. This definition is justified because, based on the cognitive competence and memory function of older adults, if such individuals do not use the Internet for one month, then their likelihood of doing so again decreases. However, the specific definition of Internet withdrawal among older adults used in the literature needs to be explored more in the future.

A critical role of technology as an important health promotion strategy for older adults in low- and middle-income countries has been proposed [49]. Our study on factors associated with Internet adoption and withdrawal provides a new lens that can help health educators to understand strategies that foster older adults in learning, an important element for active aging. Specifically, this study examining factors and reasons associated with Internet adoption and withdrawal from nationally representative data on middle-aged and older adults in Taiwan found that the learning motivation and learning models for older adults may not be different by gender, but to prevent withdrawal from learning by middle-aged and older adults, it is important that the approach be different by gender. With our findings that women were more likely to be affected by health and anthropic factors or accidental barriers, we suggest that in addition to focusing on health barriers, such as relearning to overcome cognitive declines, it is also important to work to reduce anthropic factors or accidental barriers, possibly by providing more resources, to encourage women to take more learning opportunities. In addition, for health educators to design courses and activities, it is essential that learning satisfies older adults’ needs, such as keeping up with the world or job needs, such that they gain the knowledge and skills that could increase quality of life and assist them to transition into aging successfully.
